# Modified Sodium hyaluronate conjugated to riboflavin (Har® 0.1 %) as lubricant eyedrops in the treatment of dry eye: A prospective randomised study

**DOI:** 10.1016/j.heliyon.2024.e35527

**Published:** 2024-07-31

**Authors:** Ciro Caruso, Luca D'Andrea, Michele Rinaldi, Ivana Senese, Raffaele Piscopo, Ciro Costagliola

**Affiliations:** a Corneal Transplant Center, Pellegrini Hospital, Naples, Italy; b Department of Neurosciences, Reproductive Sciences and Odontostomatology, University of Naples “Federico II”, Department of Public Health, Naples, Italy; c Department of Neurosciences, Reproductive Sciences and Odontostomatology, University of Naples “Federico II”, Naples, Italy

## Abstract

**Background:**

This study evaluates the therapeutic efficacy of HAr® (a novel ophthalmic solution containing modified hyaluronic acid covalently linked to riboflavin) compared to hyaluronic acid eye drops in patients with dry eye disease (DED).

**Methods:**

Sixteen consecutive patients with bilateral medium to severe DED were divided into two groups. Group 1 received HAr® 0.1 % (Ribohyal®), while Group 2 received HA 0.1 % eye drops. Parameters such as Ocular Surface Disease Index (OSDI) score, osmolarity, break-up time (BUT), non-invasive BUT (NIBUT), tear meniscus measurement, Schirmer test, and Oxford Staining were evaluated. This study has been successfully registered on ClinicalTrials.gov public (Identifier NCT06122428)

**Results:**

The Ribohyal group showed faster improvement in OSDI scores, with a statistically significant difference at 2 h (mean classification difference: −51.75; p = 0.0003). Photophobia significantly reduced at 2 h, 4 weeks, and 8 weeks in the Ribohyal group compared to baseline (p < 0.0001). Osmolarity improved significantly after 8 weeks in both groups (p < 0.0001).

**Conclusions:**

HAr® 0.1 % (Ribohyal®) effectively reduced DED symptoms and improved photophobia within 2 h of instillation, lasting up to 8 weeks.

## 1 Introduction

Dry eye disease (DED) is now recognized as a multifactorial condition that affects the tear film, leading to tear film instability, cellular hyperosmolarity, apoptosis of conjunctival and corneal cells, ocular inflammation, and impaired vision quality [[1], [2], [3], [4]]. DED symptoms, such as ocular discomfort and photophobia, can be induced or worsened by environmental agents, such as UV radiation, or iatrogenic factors, such as cataract surgery [[5], [6], [7], [8]]. Patients affected from dry eye disease can have significantly impacted their quality of life and daily activities, with a possible permanent need of eyedrops to manage their DED [9,10]. Hyaluronic acid (HA), a naturally occurring polysaccharide, has water-retaining properties, it promotes ocular surface hydration and helps to restore the quantity and quality of the tear film [11,12]. HA lubricating properties allows it to remain on the ocular surface for an extended duration, thus HA-based artificial tears are considered the most effective therapeutic agents for dry eye disease [13].

Studies have shown that riboflavin-based formulations of eye drops can provide protection against UV radiation and help prevent excessive tear film evaporation and instability, which is a key factor in the development of dry eye disease [14,15]. Sodium hyaluronate covalently conjugated to riboflavin, specifically HAr® 0.1 % (Ribohyal®), is a novel ophthalmic solution that aims to optimize ocular hydration and enhance ocular surface comfort.

This study aimed to evaluate the efficacy and safety of Ribohyal® artificial tear in treating ocular discomfort and improving tear film stability in patients with dry eye disease. The primary objective of this study was to evaluate the efficacy and safety of Ribohyal® (HAr® 0.1 %) in patients with dry eye disease (DED). The secondary objective was to compare its effectiveness with standard HA eye drops. The study initially included 18 patients to account for possible drop-outs, ensuring the final analysis would have sufficient power. Various tests, such as the Schirmer test, break-up time, lacrimal meniscus height, and meibography, are commonly used to assess tear film alterations. Unfortunately, their reliability is limited by significant inter- and intra-observer variability, so there is a growing trend toward using devices with automated software [16,17]. In this study automated instruments (Tear-Check and I-Pen) were used to objectively evaluate tear film characteristics and to compare the results of Riboyal treatment with a group of DED patients using HA alone.

## 2 Methods

### 2.1 Study design

**Fig. 1:**
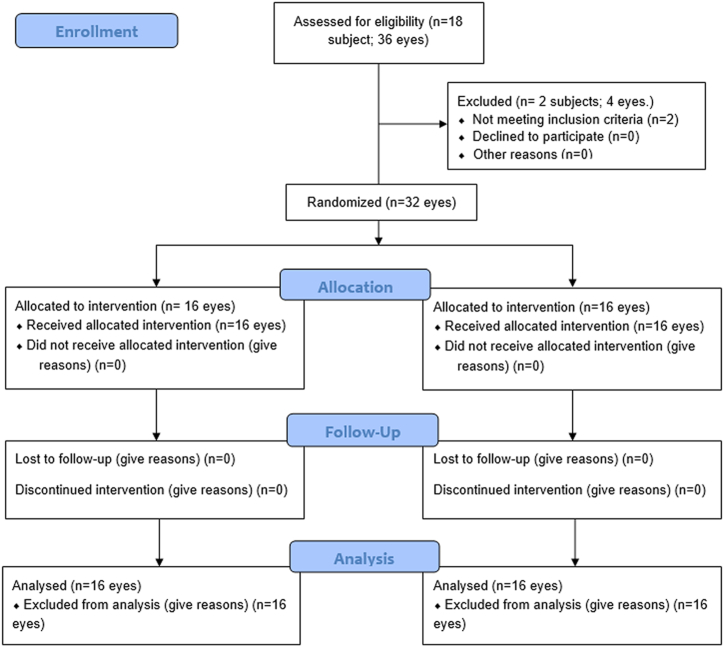
CONSORT diagram.

This is a prospective, double-blind, interventional, randomized, comparative study on 32 eyes from 16 patients (7 males and 9 females). All the patients were enrolled at the University of Naples Federico II from February 2023 to July 2023, with 18 patients initially recruited to account for possible drop-outs, but two were excluded due to not meeting the inclusion criteria (Fig. 1). The study was approved by the institution's review board (Comitato Etico Campania Centro - Asl Napoli 1, aut. n. 1269/2020) and was conducted in accordance with good clinical practice and complied with the principles outlined in the Declaration of Helsinki. This study has been successfully registered on ClinicalTrials.gov public (Identifier NCT06122428). All participants provided signed informed consent.

The 32 eyes enrolled in the study were randomized into two groups.

Group *1*-*experimental*: composed of 16 eyes (8 patients) assigned for the use of modified hyaluronic acid, Ribohyal®.

Group *2*-*control*: composed of 16 eyes (8 patients) assigned for the use of Hyaluronic acid 0.1 % alone (Control Group).

For the assignment to the two groups (group 1-experimental/Ribohyal® group-, and Group 2 -Control group-), a random assignment rule was created from a software and generated for each patient. Randomization was performed using GraphPad Prism software, which generated random assignment for each patient. Blinding was ensured by labeling the eye drop bottles with only numbers (1 for Ribohyal® and 2 for HA 0.1 %) and ensuring that both the patients and the operators administering the drops or assessing the patients were unaware of the group assignments. After the randomization, the patients were instructed to use the corresponding eye drops labeled with only a number (1- Riboyal eyedrops for Group 1 and 2- HA 0.1 % eyedrops for Group 2) based on their assigned group. The number of eye drop instillations prior to treatment was not defined to reflect real-world usage patterns and patient adherence. This approach allows the study to capture the variability in patient behavior and provides a more accurate assessment of the treatment's effectiveness. The operators administering the eye drops or checking the patients at the visits were also unaware of the group from which each patient had been assigned. The patients were instructed to use the assigned eye drops choosing from two different frequency options: *no more than 3\4 times a day or no more than 5\6 times a day*, according to their needs.

A special form was provided to all patients to record the daily frequency of instillation.

After being included in the study, all parameters were evaluated at three time points: the first base line visit (T0), four weeks after treatment (T1-4w), and eight weeks after treatment (T2-8w).

In addition, after the initial visit at T0, all patients remained in the clinic for 2 h after instillation and then OSDI data were collected and recorded (T2h). The evaluations were conducted both clinically and instrumentally.

### 2.2 Study population

This institutional study included 16 consecutive Caucasian patients with bilateral, mild to severe DED [18], resulting in a total of 32 eyes. As per other studies published [2,3,[19], [20], [21]] inclusion criteria for patient selection included a dry eye history of at least three months, OSDI score ≥23, and positivity in one or more of the following established diagnostic criteria.

- *Tear Break-Up Time (BUT) <7* *s*
- *Schirmer test I at 5* *min* < 10 mm
- *Positivity of the ocular surface epithelium to vital staining based on the Oxford scheme* [22].
- *Tear osmolarity> 308 mOsm/L or a difference of at least 8 mOsm/L between both eyes.*

The exclusion criteria considered, as reported in previously published papers [2,19], were.

- *Age below 18 years*
- *Severe pre-existing ocular surface affections*
- *Unilateral dry eye syndrome*
- *Refractive surgery performed within the last six months*
- *Eye surgery performed within the last three months*
- *Previous herpetic keratitis*
- *Signs of active corneal infection*
- *Systemic or topical steroid therapy within the last 30 days*
- *Any topical therapy within the last 14 days*
-Inability to understand the informed consent.

### 2.3 Outcome measures

The main outcomes were assessed objectively with a slit lamp or with the help of automated software (Tear-check and I-Pen) analyzing several lacrimal tear film features (Osmolarity, BUT, and NIBUT) and subjectively using questionnaires (OSDI) already validated.

For each patient, the following parameters were evaluated.

- *OSDI score (total OSDI score);*
- *Photophobia (as rated in the OSDI score);*
- *Osmolarity;*
- *Break-Up Time (BUT);*
- *Non-invasive BUT (NIBUT);*
- *Central tear meniscus height on the inferior eyelid (TMH);*
- *Schirmer test (ST);*
- *Oxford scheme staining* [22]- assessed with 2 μl of 2 % sterile fluorescein instilled into each conjunctival sac with a micro-pipette (using a sterile tip);

TMH and NIBUT measurements were conducted using Tear-check automated software (I-MED Pharma Inc, Dollard-des-Ormeaux, Quebec, Canada), whereas osmolarity measurements were performed using I-Pen (I-MED Pharma Inc, Dollard-des-Ormeaux, Quebec, Canada) [23].

The tests were performed in each eye, following a specific order.

1. Tear osmolarity measurement
2. Tear Break-Up Time (BUT) test
3. Biomicroscopic observation with white light and cobalt blue light after instillation 2 μl of 2 % sterile fluorescein to evaluate corneal staining according to the Oxford scheme.
4. Schirmer test I (without anesthetic) at 5 min after a 20-min interval.

Later, additional tests were performed using the Tear Check device, evaluating the following parameters.

5. TMH
6. Non-invasive Break-Up Time (NIBUT)

Photophobia was the primary outcome based on which we calculated the sample size. Based on the data present in our cohort database, hyaluronic acid has minimal to no effect on photophobia; therefore, we expected that Ribohyal treatment would reduce the photophobia score by at least 45 % at 4 weeks. To detect this reduction with a power of 80 %, we needed six observations per group for a total of 12 observations. We included 18 to account for possible drop-outs. A first follow-up visit (T1-4w_Ribohyal/Control) was conducted four weeks later, including evaluations of osmolarity, OSDI, BUT, NIBUT, corneal staining, Schirmer test, and additional Tear Check evaluations.

Four weeks after the first follow-up visit, the final evaluation (T2-8w_Ribohyal/Control) was conducted, repeating the same diagnostic tests performed at T0 and T1 in the same sequence.

All data collected were reported, processed, and subjected to statistical analysis. The contents of the eye drops used in the treatment were revealed to the operators at a later time.

### 2.4 Statistical analysis

Statistical analysis was performed using Prism 9 software from GraphPad Software, LLC. One-Way ANOVA corrected with Dunn's multiple comparisons tests was employed to evaluate the effect of each eye drop on the different parameters examined and to assess the possible differences in efficacy between the two treatment groups. Fisher's exact test was conducted to determine if there was a significant difference in instillation frequency between the two groups. A p-value less than 0.05 was considered statistically significant.

## 3 Results

The patients enrolled were 16 (7 males and 9 females), with a total of 32 eyes. The baseline data are summarized in Table 1. The simple size recruitment was reached with no losses, exclusion, and/or reported harms. The trial ended 3 months after the randomization.

**Table 1:** Baseline data.

Group	Experimental l Control			
	**Mean**	**SEM**	**Mean**	**SEM**
**OSDI**	31.250	1.740	30.500	1.954
**Photophobia**	2.750	0.112	2.625	0.125
**Osmolarity**	320.938	2.199	319.438	1.966
**BUT**	4.375	0.407	4.938	0.281
**NIBUT**	3.525	0.406	4.275	0.316

**Fig. 2:**
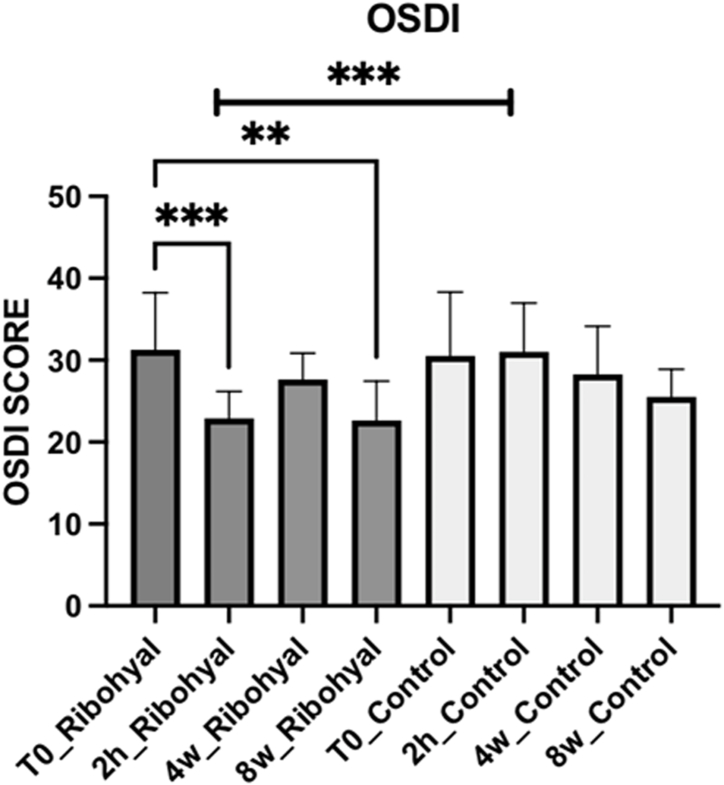
OSDI score. One-Way Anova corrected Dunn's multiple comparisons test. *P value < 0.05; **P value < 0.01; ***P value < 0.001.

*OSDI SCORE*: After eight weeks treatment, both groups showed a statistically significant decrease in OSDI score without a significant difference between the two groups. This indicates that the long-term effectiveness in terms of OSDI score did not differ significantly between the Ribohyal® group and the Control group (See Table 2 and Fig. 2). However, OSDI score at 2 h in Riboyal group (T2h_Ribohyal) had a statistically significant difference when compared to the Control group (T2h_Control), with a mean ranking difference of −51.75 and a p-value of 0.0003 this suggests a faster effect of the Ribohyal®. *PHOTOPHOBIA:* In the Ribohyal® group the photophobia, if compared to the initial visit (T0), showed a statistically significant reduction at the 2 h, 4 weeks, and 8 weeks visits with a p-value of less than 0.0001. This suggests that patients in the Ribohyal group experienced a significant improvement in photophobia throughout the study period.

**Table 2:** OSDI score.

Dunn's multiple comparisons test	Mean rank diff	Significance	Summary	P Value
T0_Ribohyal vs.T0_Control	7188	No	Ns	>0,9999
2 h_Ribohyal vs.2 h_Control	−51,75	Yes	***	0,0003
4w_Ribohyal vs.4w_Control	1875	No	Ns	>0,9999
8w_Ribohyal vs.8w_Control	−17,06	No	Ns	0,7671

**Test details**	**Mean rank 1**	**Mean rank 2**	**Mean rank difference**	**n1**	**n2**	**Z**
T0_Ribohyal vs.T0_Control	85,69	78,50	7188	16	16	0,5499
2 h_Ribohyal vs.2 h_Control	34,81	86,56	−51,75	16	16	3959
4w_Ribohyal vs.4w_Control	70,63	68,75	1875	16	16	0,1434
8w_Ribohyal vs.8w_Control	37,00	54,06	−17,06	16	16	1305

**Fig. 3:**
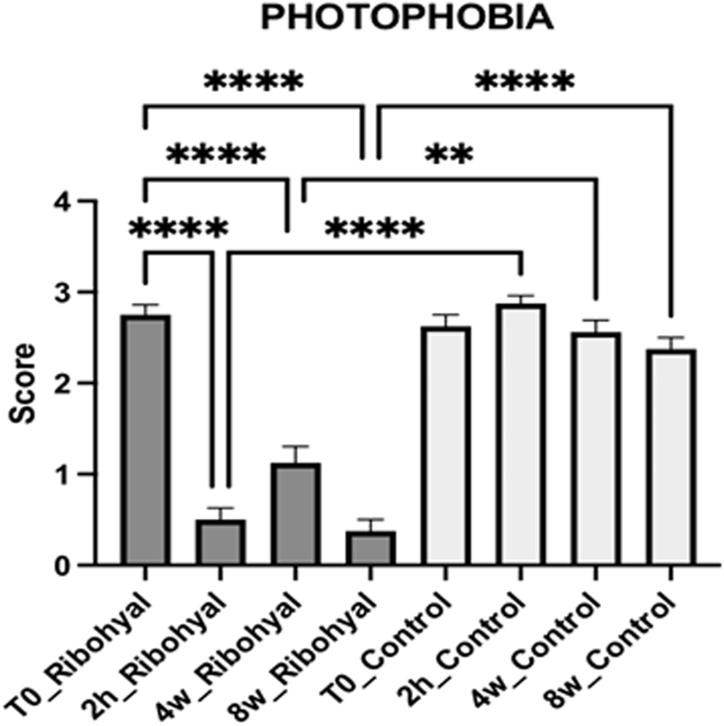
Photophobia score. One-Way Anova corrected Dunn's multiple comparisons test. *P value < 0.05; **P value < 0.01; ***P value < 0.001; ****P value < 0.0001.

Furthermore, when comparing the Ribohyal® group to the Control group, the Ribohyal® group showed a statistically significant difference in photophobia improvement at the 2 h, 4 weeks, and 8 weeks visits. The p-values were less than 0.0001 at the 2-h mark and after 8 weeks, and p = 0.0014 after 4 weeks. This indicates that the Ribohyal® group exhibited a significantly greater reduction in photophobia compared to the Control group at these time points (Fig. 3).

**Fig. 4:**
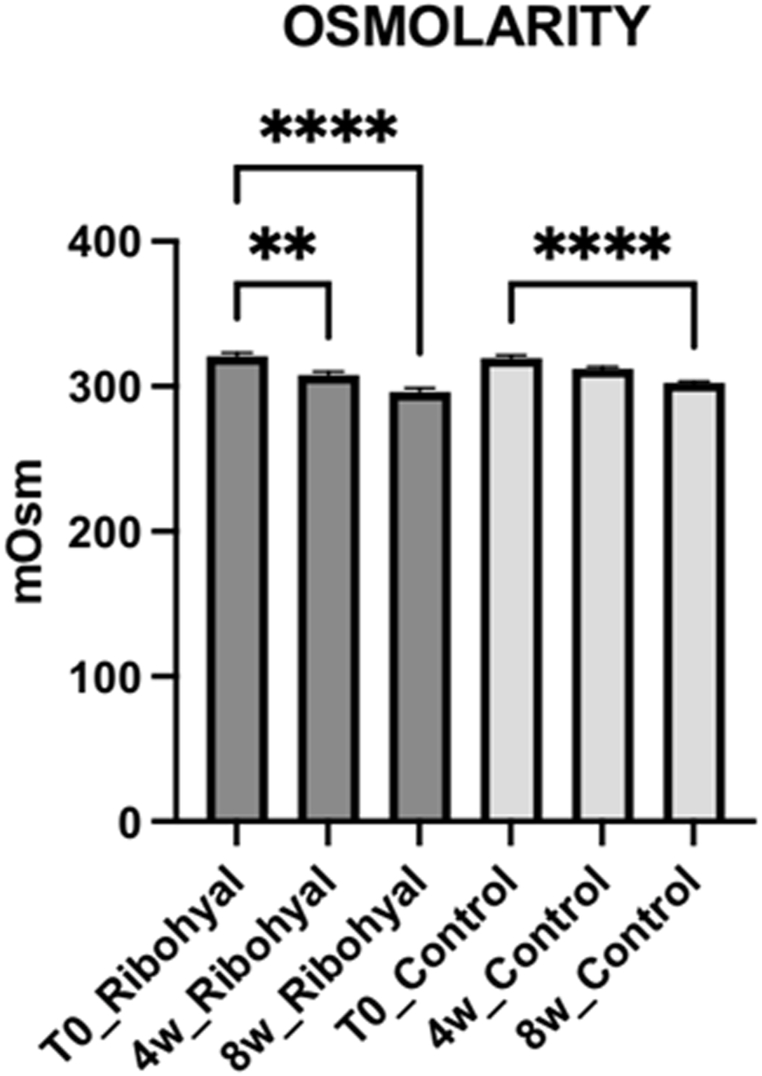
OSMOLARITY. One-Way Anova corrected Dunn's multiple comparisons test. *P value < 0.05; **P value < 0.01; ***P value < 0.001; ****P value < 0.0001.

**Fig. 5:**
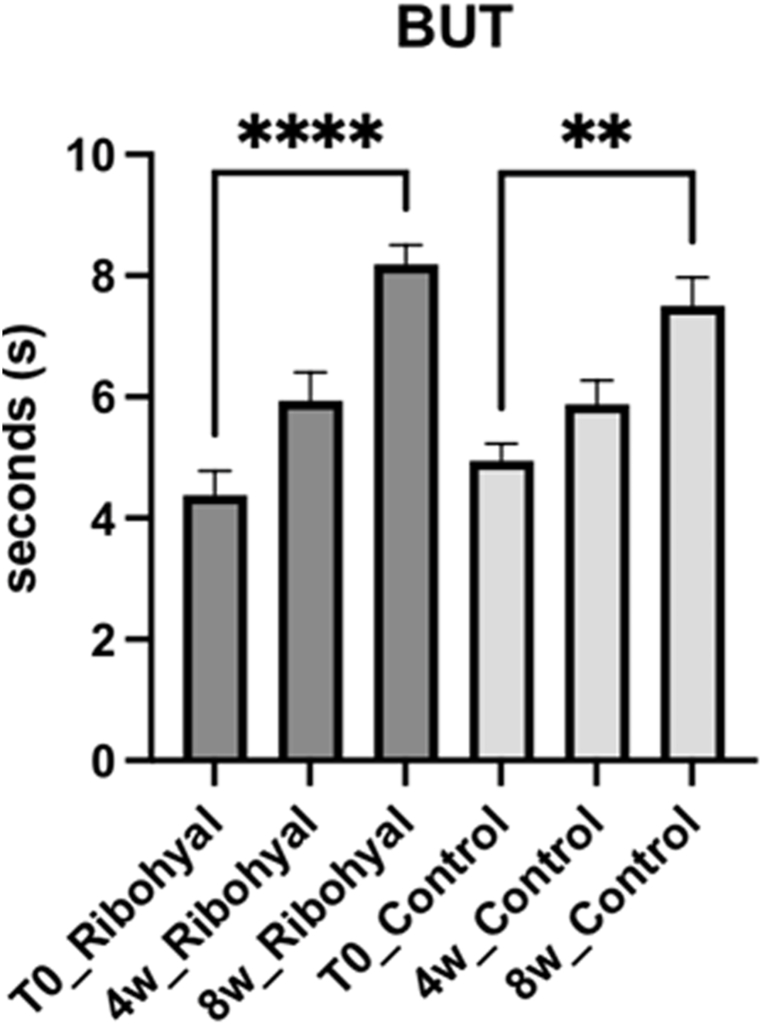
BUT. One-Way Anova corrected Dunn's multiple comparisons test * P value < 0.05; **P value < 0.01; ***P value < 0.001; ****P value < 0.0001.

**Fig. 6:**
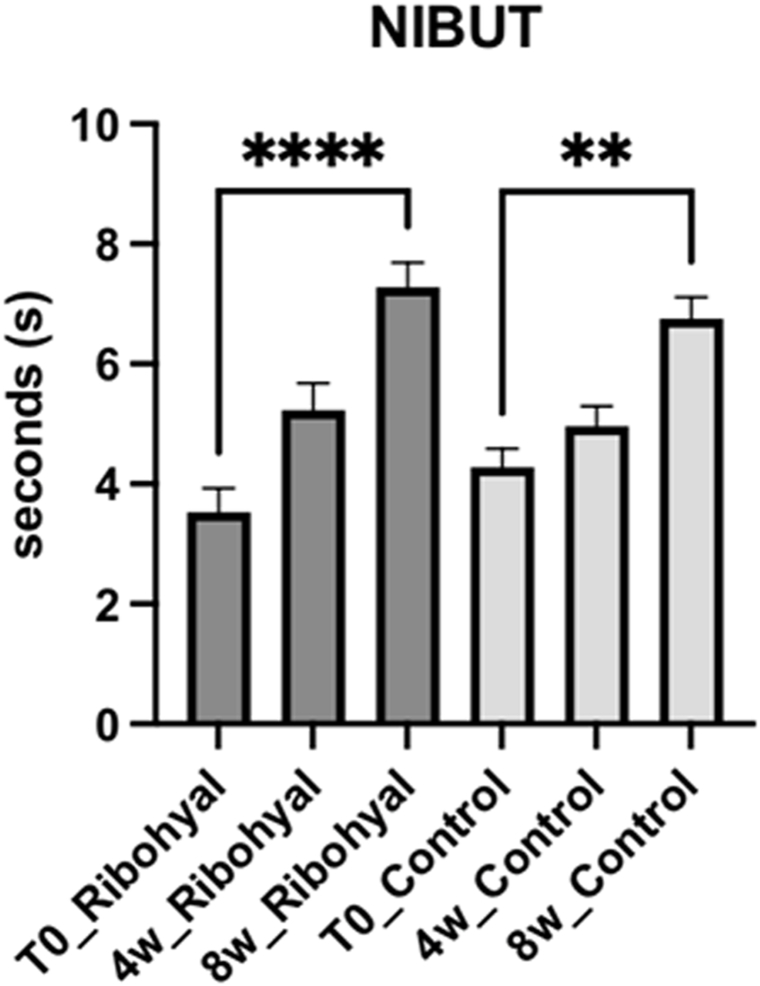
NIBUT. One-Way Anova corrected Dunn's multiple comparisons test. *P value < 0.05; **P value < 0.01; ***P value < 0.001; ****P value < 0.0001.

*OSMOLARITY:* When compared to baseline, both the Ribohyal® group and the control group showed a statistically significant improvement in the osmolarity after 8 weeks of treatment (p < 0.0001), with no significative difference between the two groups. This indicates that both treatment approaches were effective in reducing osmolarity levels in patients with dry eye disease (Fig. 4). *BUT AND NIBUT:* Concerning the parameters BUT and NIBUT, they significantly improved in both the Ribohyal® (p < 0.001 and p < 0.0001) and control group (p < 0.0015 and p < 0.0016), and they were not significantly different from each other. In the Ribohyal® group, BUT showed a p-value of less than 0.0001, while in the control group, the p-value was 0.0014. Similarly, for NIBUT, the Ribohyal® group had a p-value of less than 0.0001, and the control group had a p-value of 0.0015. However, no significant difference was observed between the Ribohyal® group and the Control group for these parameters. These results suggest that both treatment groups experienced significant improvements in tear film stability (Fig. 5, Fig. 6).

*OXFORD SCHEME, SCHIRMER TEST, LACRIMAL MENISCUS HEIGHT:* In both groups, an overall improvement in Oxford scale and lacrimal meniscus was shown without a statistically significant difference between the two groups. Regarding the Schirmer test, a statistically significant improvement was shown for both groups, which increased from 5,44 at T0 to 7,69 at T1-4w, to 10,63 at T2-8w in the Ribohyal® group, and an increase from 6,19 at T0 to 9,44 at T1-4w to 11,06 at T2-8w in the control group. ADVERSE EFFECTS: No significant adverse effects were observed in either group during the study. All patients tolerated the treatments well, with no reports of serious ocular or systemic side effects.

*INSTILLATION FREQUENCY:* In the Ribohyal® group, the instillation frequency was reported as 71.43 % for 3/4 times a day and 12.50 % for 5/6 times a day. On the other hand, in the Control group, the instillation frequency was reported as 28.57 % for 3/4 times a day and 87.50 % for 5/6 times a day. These differences in instillation frequency between the two groups were found to be statistically significant with a p-value of less than 0.01 (p < 0.01). This suggests that there was a significant variation in the frequency of eye drop instillation between the Ribohyal® group and the control group, with a higher proportion of patients in the Ribohyal® l group using the eye drops 3/4 times a day and a higher proportion of patients in the Control group using the eye drops 5/6 times a day (Table 3).

**Table 3:** Total Percentage of row.

Total percentage of row	3/4 time day	5/6 time a day
Ribohyal experimental group	71.43 %	28.57 %
Control group	12.50 %	87.50 %

## 4 Discussion

DED is a multifactorial condition affecting different features of the tear film and its treatment is addressed to improve several components involved in tear homeostasis [3,6,24].

The experimental question beyond this study is to test if a HA conjugated with riboflavin (Ribohyal®) can be an effective and a tolerated option in patients suffering from DED. If hyaluronic acid use is a milestone in the treatment of DED [3], several studies have experimented if its effect could have been improved adding other molecules to the same pharmaceutical formulation [9,[24], [25], [26]]. Nowadays, the rationale beyond the use of conjugated riboflavin is its role in the protection from the UVA oxidative damage [14], as numerous studies have demonstrated the potential role of oxidative damage in the development of DED [[27], [28], [29]]. Previous studies have demonstrated the protective role of riboflavin against UV-induced oxidative damage, which is a contributing factor in the pathogenesis of dry eye. Di Nezza et al. [14] showed that riboflavin could reduce oxidative stress on the ocular surface, thereby preventing damage to the tear film and conjunctival cells. Vizzarri et al. [15] demonstrated the efficacy of a riboflavin-based formulation in reducing tear evaporation and improving tear film stability. These findings support our results, where the Ribohyal® group showed significant improvement in photophobia and other tear film parameters, indicating the efficacy of riboflavin in the management of DED.

The main outcomes were assessed, objectively, with a slit lamp or with the help of automated software (Tear-check and I-Pen) analyzing several lacrimal tear film features (Osmolarity, BUT, and NIBUT), and subjectively, using questionnaire (OSDI) already validated [10,21]. Research by Di Cello et al. [17] demonstrated that the use of advanced diagnostic tools like the I-Med Tear Check provides more reliable and objective measurements of tear film characteristics compared to traditional methods. These instruments reduce inter- and intra-observer variability, offering a more precise evaluation of treatment efficacy in dry eye disease.

All the results coming from Ribohyal® group were statistically analyzed and compared to those coming from another group of patients that used HA 0.1 % alone (Control group).

According to the results of this study, both groups showed improvement in the objectively evaluated parameters. If compared to baseline, Osmolarity, BUT, NIBUT and Schirmer test exhibited statistically significant improvements in both groups. When compared to the HA alone group, the Ribohyal® group showed a mildly superior efficacy in improving osmolarity, with a greater reduction of 24 mmOsm, compared to 23 mmOsm, respectively (p < 0.05). However, no statistically significant improvements were found in the other evaluated parameters (Oxford rating scale, lacrimal meniscus height) within or between the two groups. Another difference found was that, when compared to the Control group, Ribohyal® group patients experienced a greater reduction in OSDI score at 2 h after the first administration and also a less need of eye drops instillation frequency: indeed there was a significant variation in the frequency of eye drop instillation between the Ribohyal® group and the Control group, with a higher proportion of patients in the Ribohyal® l group using the eye drops 3/4 times a day and a higher proportion of patients in the Control group using the eye drops 5/6 times a day.

According to us, this may be attributed to the biochemical modification in HA done through the stable covalent link between riboflavin and HA (Ryboyal).

HA, a vital component of the extracellular matrix in connective tissue, has a high hydrophilicity, a large molecular weight and its degradation goes through hydrolysis by hyaluronidases [12,24,30].

Hyaluronic acid (HA) is a vital component of the extracellular matrix in connective tissue, known for its high hydrophilicity and large molecular weight. Its degradation occurs through hydrolysis by hyaluronidases. To enhance the stability and preserve the fundamental characteristics of HA for clinical applications, two methods of modification—derivatization and cross-linking—can be employed. These modifications involve reactions with the functional groups present in HA (-COOH and –OH). For example, the hydroxyl groups (-OH) of HA chains react with divinyl sulfone (DVS), a cross-linking agent, under alkaline conditions to form stable hydrogels with sulfonyl-bisethyl linkages. Increasing the number of these bonds enhances mechanical stability and resistance to enzymatic degradation. [31] Similarly, 1,4-butanediol diglycidyl ether can be used as a cross-linking agent to stabilize HA, resulting in strong hydrogels with favorable viscoelastic characteristics. [32] These cross-linked forms of HA follow the same metabolic degradation pathways as unmodified HA. The modified hyaluronic acid in Ribohyal® 0.1 % is synthesized by covalently binding riboflavin to HA's COOH and OH groups. This biochemical modification maintains the mechanical properties of HA while supporting better enzymatic degradation by reducing the functional groups susceptible to hyaluronidase. Consequently, Ribohyal® provides prolonged residence time on the ocular surface, enhancing its effectiveness in treating dry eye disease. However, after eight weeks of treatment, both groups showed a significant decrease in OSDI score without a significant difference between the two groups. Among the different OSDI items, a specific attention was paid on photophobia as this is related to UV exposure, which in turn is contrasted by the riboflavin UV blocking effect [15,33,34].

The combined use of riboflavin and HA in Ribohyal® enhances ocular surface protection and hydration. Riboflavin, known for its UV-blocking effect, prevents oxidative damage, a significant factor in DED pathophysiology. This was evident from the significant improvement in photophobia in the Ribohyal® group, which can be attributed to riboflavin's protective mechanism. HA, on the other hand, is known for its hydrophilic properties, enhancing tear film stability. The improvement in parameters such as osmolarity, BUT, and NIBUT in both groups highlights HA's role in maintaining tear film stability, while the superior results in the Ribohyal® group underscore the added benefits of riboflavin.

Among all the ocular discomfort parameters rated by the patient in OSDI questionnaire, only photophobia showed statistically significative differences: in the Ribohyal® group, the photophobia, when compared to the initial visit (T0), showed a statistically significant reduction at 4 weeks, and 8 weeks visits (p*-*value of less than 0.0001). Furthermore, when comparing the Ribohyal® group to the Control group, the Ribohyal® group showed a statistically significant difference in photophobia improvement at 4 weeks, and 8 weeks visits (with a p-value of less than 0.0001 at 8 weeks check, and p = 0.0014 after 4 weeks check).

It can be hypothesized that the reason is the well-documented riboflavin anti-UV effect and oxidative damage protection, as it has shown that cumulative oxidative damage affects corneal and conjunctival epithelial cells, resulting in alterations in goblet cell density, lacrimal gland structure and secretory activity, with a damage to the Meibomian glands [[28], [29], [30]]. This aligns with previous research [14] confirming the additive protection in the DED pathophysiology provided by riboflavin as an antioxidant agent.

## 5 Conclusions

This study has confirmed the effectiveness of both HA and Ribohyal® in significantly improving several lacrimal film parameters. Moreover, relying on the modifications in the OSDI score before and after the treatment, both the drugs were well tolerated. If compared to HA, the modified form of HA covalently bonded to riboflavin, appears to be more effective in specific aspects, so that it can be considered as an alternative treatment for DED, particularly in those patients performing external activities or exposed to a higher UV-related oxidative damage.

Despite the promising results, this study has several limitations. The sample size was relatively small, which might limit the generalizability of the findings. The study duration was also short, and longer-term effects of Ribohyal® could not be assessed. Future research with larger sample sizes, longer follow-up periods are recommended. Additionally, further studies specifically addressed to DED patients highly exposed to UV-rich environments could be beneficial in order to understand the impact of UV protection on DED prevention and treatment.

## Funding

This research received no external funding.

## Ethics and informed consent

The study was approved by the institution's review board (Comitato Etico Campania Centro - Asl Napoli 1, aut. n. 1269/2020) and was conducted in accordance with good clinical practice and complied with the principles outlined in the Declaration of Helsinki. This study has been successfully registered on ClinicalTrials.gov public (Identifier NCT06122428). All participants provided signed informed consent.

## Data availability statement

The data associated with this study have not been deposited into a publicly available repository. However, the data will be made available upon request. Researchers interested in accessing the data can contact the corresponding author for further information.

## CRediT authorship contribution statement

**Ciro Caruso:** Writing – review & editing, Project administration, Formal analysis, Data curation, Conceptualization. **Luca D'Andrea:** Writing – review & editing, Writing – original draft, Project administration, Conceptualization. **Michele Rinaldi:** Supervision. **Ivana Senese:** Methodology, Investigation, Conceptualization. **Raffaele Piscopo:** Writing – review & editing, Writing – original draft, Project administration, Methodology, Investigation. **Ciro Costagliola:** Supervision.

## Declaration of competing interest

The authors declare that they have no known competing financial interests or personal relationships that could have appeared to influence the work reported in this paper.
