# Supplementary material for: Modified Sodium hyaluronate conjugated to riboflavin (Har® 0.1 %) as lubricant eyedrops in the treatment of dry eye: A prospective randomised study
Source: Heliyon. 2024 Jul 31;10(15):e35527. doi: 10.1016/j.heliyon.2024.e35527 (PMC11336708; doi:10.1016/j.heliyon.2024.e35527)
Supplement: Multimedia component 1 [file mmc1.pdf]

CONSORT Harms 2022 integrated into CONSORT 2010 items checklist of information to include when reporting a randomised trial

Section/Topic	Item No	Checklist item	Reported on page No
**Title and abstract**			
	1a	Identification as a randomised trial in the title	1
	1b	Structured summary of trial design, methods, results of outcomes of benefits and harms, and conclusions (for specific guidance see CONSORT for abstracts)	1
**Introduction**			
Background and objectives	2a	Scientific background and explanation of rationale	2
	2b	Specific objectives or hypotheses for outcomes benefits and harms	2
**Methods**			
Trial design	3a	Description of trial design (such as parallel, factorial) including allocation ratio	4
	3b	Important changes to methods after trial commencement (such as eligibility criteria), with reasons	4-5
Participants	4a	Eligibility criteria for participants	4-5
	4b	Settings and locations where the data were collected	4
Interventions	5	The interventions for each group with sufficient details to allow replication, including how and when they were actually administered	4
Outcomes	6a	Completely defined pre-specified primary and secondary outcome measures for both benefits and harms, including how and when they were assessed	5-6
	6b	Any changes to trial outcomes after the trial commenced, with reasons	NO CHANGES
	6c	Describe if and how non-prespecified outcomes of benefits and harms were identified, including any selection criteria, if applicable	6
Sample size	7a	How sample size was determined	6
	7b	When applicable, explanation of any interim analyses and stopping guidelines	NOT APPLICABLE

Scansionato con CamScanner

Section/Topic	Item No	Checklist item	Reported on page No
Randomisation:			
Sequence generation	8a	Method used to generate the random allocation sequence	4
	8b	Type of randomisation; details of any restriction (such as blocking and block size)	5
Allocation concealment mechanism	9	Mechanism used to implement the random allocation sequence (such as sequentially numbered containers), describing any steps taken to conceal the sequence until interventions were assigned	4
Implementation	10	Who generated the random allocation sequence, who enrolled participants, and who assigned participants to interventions	4
Blinding	11a	If done, who was blinded after assignment to interventions (e.g., participants, care providers, those assessing outcomes of benefits and harms) and how	4
	11b	If relevant, description of the similarity of interventions	NOT RELEVANT
Statistical methods	12a	Statistical methods used to compare groups for primary and secondary outcomes of both benefits and harms	6
	12b	Methods for additional analyses, such as subgroup analyses and adjusted analyses	5
**Results**			
Participant flow (a diagram is strongly recommended)	13a	For each group, the numbers of participants who were randomly assigned, received intended treatment, and were analysed for outcomes of benefits and harms	7
	13b	For each group, losses and exclusions after randomisation, together with reasons	7
Recruitment	14a	Dates defining the periods of recruitment and follow-up for outcomes of benefits and harms	4
	14b	Why the trial ended or was stopped	7
Baseline data	15	A table showing baseline demographic and clinical characteristics for each group	7
Numbers analysed	16	For each group, number of participants (denominator) included in each analysis and whether the analysis was by original assigned groups and if any exclusions were made	7
Outcomes and estimation	17a	For each primary and secondary outcome of benefits and harms, results for each group, and the estimated effect size and its precision (such as 95% confidence interval)	7
	17a2	For outcomes omitted from the trial report (benefits and harms), provide rationale for not reporting and indicate where the data on omitted outcomes can be accessed	NOT APPLICABLE
	17b	Presentation of both absolute and relative effect sizes is recommended, for outcomes of benefits and harms	
	17c	Report zero events if no harms were observed	7
Ancillary analyses	18	Results of any other analyses performed, including subgroup analyses and adjusted analyses, distinguishing pre-specified from exploratory	7
Harms	19	All important harms or unintended effects in each group (for specific guidance see CONSORT for harms)	7-10

Scansionato con CamScanner

Section/Topic	Item No	Checklist item	Reported on page No
**Discussion**			
Limitations	20	Trial limitations, addressing sources of potential bias related to the approach to collecting or reporting data on harms, imprecision, and, if relevant, multiplicity or selection of analyses	11
Generalisability	21	Generalisability (external validity, applicability) of the trial findings	11
Interpretation	22	Interpretation consistent with results, balancing benefits and harms, and considering other relevant evidence	11
**Other information**			
Registration	23	Registration number and name of trial registry	4
Protocol	24	Where the full trial protocol and other relevant documents can be accessed, including additional data on harms	
Funding	25	Sources of funding and other support (such as supply of drugs), role of funders	11

Note: Adapted from Schulz (2010) to integrate items of CONSORT Harms 2022 (Junqueira 2022) [https://creativecommons.org/licenses/by/2.0/]. CONSORT items 1b, 2b, 6a, 11a, 12a, 13a, 14a, 16a, 17a, 17b, 18, 20 and 24 of were modified to incorporate elements relevant to the reporting of harms. Two new items were added (item 6c and 17a2). Please see the CONSORT Harms 2022 statement for additional details (Junqueira 2022).

We strongly recommend reading the CONSORT 2010 statement (Schulz 2010) in conjunction with the CONSORT Harms 2022 statement (Junqueira 2022) for important clarifications on all the items. If relevant, we also recommend reading CONSORT extensions for cluster randomised trials, non-inferiority and equivalence trials, non-pharmacological treatments, adaptive designs, pilot and feasibility studies, multi arm trials, cross-over and pragmatic trials. Additional extensions are forthcoming: for those and for up-to-date references relevant to this checklist, see EQUATOR Network.

References

Junqueira DR, Zorzela L, Golder S, Loke Y, Gagnier JJ, Julious SA, Li T, Mayo-Wilson E, Pham B, Phillips R, Santaguida P, Scherer RW, Gøtzsche PC, Moher D, Ioannidis JPA and Vohra S on behalf of the CONSORT Harms Group. CONSORT Harms 2022 statement, explanation, and elaboration: updated guideline for the reporting of harms in randomised trials. *BMJ* 2023 **381:** e073725 DOI 10.1136/bmj-2022-073725

Schulz KF, Altman DG, Moher D, for the CONSORT Group. CONSORT 2010 Statement: updated guidelines for reporting parallel group randomised trials. *BMJ* 2010 **340:**c332 doi: 10.1136/bmj.c332.

Scansionato con CamScanner

CONSORT 2010 checklist of information to include when reporting a randomised trial*

Section/Topic	Item No	Checklist item	Reported on page No
**Title and abstract**			
	1a	Identification as a randomised trial in the title	1
	1b	Structured summary of trial design, methods, results, and conclusions (for specific guidance see CONSORT for abstracts)	1
**Introduction**			
Background and objectives	2a	Scientific background and explanation of rationale	2
	2b	Specific objectives or hypotheses	2
**Methods**			
Trial design	3a	Description of trial design (such as parallel, factorial) including allocation ratio	4
	3b	Important changes to methods after trial commencement (such as eligibility criteria), with reasons	4-5
Participants	4a	Eligibility criteria for participants	4-5
	4b	Settings and locations where the data were collected	4
Interventions	5	The interventions for each group with sufficient details to allow replication, including how and when they were actually administered	4
Outcomes	6a	Completely defined pre-specified primary and secondary outcome measures, including how and when they were assessed	5-6
	6b	Any changes to trial outcomes after the trial commenced, with reasons	NO CHANGES
Sample size	7a	How sample size was determined	6
	7b	When applicable, explanation of any interim analyses and stopping guidelines	NOT APPLICABLE
Randomisation:			
Sequence generation	8a	Method used to generate the random allocation sequence	4
	8b	Type of randomisation; details of any restriction (such as blocking and block size)	5
Allocation concealment mechanism	9	Mechanism used to implement the random allocation sequence (such as sequentially numbered containers), describing any steps taken to conceal the sequence until interventions were assigned	5
Implementation	10	Who generated the random allocation sequence, who enrolled participants, and who assigned participants to interventions	4
Blinding	11a	If done, who was blinded after assignment to interventions (for example, participants, care providers, those	4

Scansionato con CamScanner

		assessing outcomes) and how	4
	11b	If relevant, description of the similarity of interventions	NOT RELEVANT
Statistical methods	12a	Statistical methods used to compare groups for primary and secondary outcomes	6
	12b	Methods for additional analyses, such as subgroup analyses and adjusted analyses	5
**Results**			
Participant flow (a diagram is strongly recommended)	13a	For each group, the numbers of participants who were randomly assigned, received intended treatment, and were analysed for the primary outcome	7
	13b	For each group, losses and exclusions after randomisation, together with reasons	7
Recruitment	14a	Dates defining the periods of recruitment and follow-up	4
	14b	Why the trial ended or was stopped	7
Baseline data	15	A table showing baseline demographic and clinical characteristics for each group	7
Numbers analysed	16	For each group, number of participants (denominator) included in each analysis and whether the analysis was by original assigned groups	7
Outcomes and estimation	17a	For each primary and secondary outcome, results for each group, and the estimated effect size and its precision (such as 95% confidence interval)	6 ~~NOT APPLICABLE~~
	17b	For binary outcomes, presentation of both absolute and relative effect sizes is recommended	6
Ancillary analyses	18	Results of any other analyses performed, including subgroup analyses and adjusted analyses, distinguishing pre-specified from exploratory	
Harms	19	All important harms or unintended effects in each group (for specific guidance see CONSORT for harms)	10
**Discussion**			
Limitations	20	Trial limitations, addressing sources of potential bias, imprecision, and, if relevant, multiplicity of analyses	11
Generalisability	21	Generalisability (external validity, applicability) of the trial findings	11
Interpretation	22	Interpretation consistent with results, balancing benefits and harms, and considering other relevant evidence	11
**Other information**			
Registration	23	Registration number and name of trial registry	4
Protocol	24	Where the full trial protocol can be accessed, if available	NOT AVAILABLE
Funding	25	Sources of funding and other support (such as supply of drugs), role of funders	12

Citation: Schulz KF, Altman DG, Moher D, for the CONSORT Group. CONSORT 2010 Statement: updated guidelines for reporting parallel group randomised trials. BMC Medicine. 2010;8:18.
© 2010 Schulz et al. This is an Open Access article distributed under the terms of the Creative Commons Attribution License (http://creativecommons.org/licenses/by/2.0), which permits unrestricted use, distribution, and reproduction in any medium, provided the original work is properly cited.

*We strongly recommend reading this statement in conjunction with the CONSORT 2010 Explanation and Elaboration for important clarifications on all the items. If relevant, we also recommend reading CONSORT extensions for cluster randomised trials, non-inferiority and equivalence trials, non-pharmacological treatments, herbal interventions, and pragmatic trials. Additional extensions are forthcoming: for those and for up-to-date references relevant to this checklist, see www.consort-statement.org.

Scansionato con CamScanner
